# Derivative estimation for longitudinal data analysis: Examining features of blood pressure measured repeatedly during pregnancy

**DOI:** 10.1002/sim.7694

**Published:** 2018-05-20

**Authors:** Andrew J. Simpkin, Maria Durban, Debbie A. Lawlor, Corrie MacDonald‐Wallis, Margaret T. May, Chris Metcalfe, Kate Tilling

**Affiliations:** ^1^ MRC Integrative Epidemiology Unit at the University of Bristol Bristol BS8 2BN UK; ^2^ Insight Centre for Data Analytics NUI Galway Ireland; ^3^ Department of Statistics Universidad Carlos III de Madrid Madrid Spain; ^4^ Population Health Sciences, Bristol Medical School University of Bristol Bristol BS8 2BN UK; ^5^ Centre for Exercise, Nutrition & Health Sciences, School for Policy Studies University of Bristol Bristol BS8 UK

**Keywords:** ALSPAC, derivative estimation, functional data analysis, longitudinal data analysis, penalized splines

## Abstract

Estimating velocity and acceleration trajectories allows novel inferences in the field of longitudinal data analysis, such as estimating change regions rather than change points, and testing group effects on nonlinear change in an outcome (ie, a nonlinear interaction). In this article, we develop derivative estimation for 2 standard approaches—polynomial mixed models and spline mixed models. We compare their performance with an established method—principal component analysis through conditional expectation through a simulation study. We then apply the methods to repeated blood pressure (BP) measurements in a UK cohort of pregnant women, where the goals of analysis are to (i) identify and estimate regions of BP change for each individual and (ii) investigate the association between parity and BP change at the population level. The penalized spline mixed model had the lowest bias in our simulation study, and we identified evidence for BP change regions in over 75% of pregnant women. Using mean velocity difference revealed differences in BP change between women in their first pregnancy compared with those who had at least 1 previous pregnancy. We recommend the use of penalized spline mixed models for derivative estimation in longitudinal data analysis.

## 1 INTRODUCTION

Linear mixed models^1^ (LMMs) are a common approach in longitudinal data analysis, with the slope—a constant estimate of change—often the key parameter of interest. When change over time is nonlinear, making inferences about this change can be problematic. Simple polynomials can describe the relationship as a combination of coefficients, but these are inflexible, and higher order polynomials are rather unstable and can lead to overfitting. Smoothing methods such as splines^2^ allow for flexible modeling of nonlinear data, and these have been adapted to repeated measure data.^3^, ^4^ Functional data analysis^5^ methods are also highly effective for modeling such data, and although some FDA approaches are designed for regularly spaced functional data, application of FPCs analysis to situations of irregular and sparse data (such as those seen in biomedical applications) is well established.^6^, ^7^, ^8^, ^9^, ^10^

When analyzing nonlinear trajectories, the rate of change of the outcome is often of primary interest, and this can be directly assessed through estimating the velocity and acceleration (ie, derivative estimation). This has been explored in the univariate case (ie, individuals measured once),^11^, ^12^, ^13^, ^14^, ^15^, ^16^, ^17^ but there has been a lack of research focused on longitudinal data (ie, individuals measured repeatedly),^18^, ^19^, ^20^ especially in the mixed model framework. A major application for derivative estimation is identifying features in individuals, such as change points in longitudinal data.^21^, ^22^, ^23^, ^24^, ^25^ When a velocity trajectory and its confidence bands^26^, ^27^, ^28^, ^29^ are fully positive, this provides evidence for an increase in the outcome measure beyond measurement error; similarly, when an acceleration trajectory and its bands are positive, this suggests that there is a change in slope, ie, a change point, in the data. Using derivative estimation, *regions* of change can be identified graphically, where traditional change point models generally provide a single estimate. This idea has been taken forward in the univariate case (ie, individuals measured once)^30^ but has not been explored in the repeated measures setting (ie, individuals measured repeatedly). More generally, derivatives can be used to estimate other features of nonlinear trajectories which capture important biological moments, including age at peak height velocity and age at adiposity rebound, which are both used as risk factors in life course epidemiology.^31^, ^32^ These applications require estimation of standard errors for velocity and acceleration trajectories, which have not previously been derived within the mixed model framework.

Another application of derivative estimation is comparing change between 2 or more groups. If change is linear, this is achieved by including a time by group interaction in the model. When change in the outcome is nonlinear over time, it follows that the interaction itself can change over time. This can be assessed by plotting the difference in mean velocity, along with confidence bands. When this difference curve and its bands are fully positive or negative, this suggests a difference in change between groups.^33^

In this article, we develop derivative estimation for 2 standard approaches—polynomial mixed models and spline mixed models. We compare their performance with an established method, principal component analysis through conditional expectation (PACE),^18^ using a simulation study. We then apply the methods to repeated blood pressure (BP) measurements in a UK cohort of pregnant women, where the goals of our analysis are to determine (i) whether and when BP change occurs (for each individual) and (ii) the association between parity and BP change (at the population level).

## 2 ESTIMATING VELOCITY AND ACCELERATION TRAJECTORIES

Consider a repeatedly measured outcome variable *y*_*ij*_, where *i* = 1, …, *n* index the *n* individuals in the sample and *j* = 1, …, *n*_*i*_ index the *n*_*i*_ observations for individual *i*, such that individuals can have different numbers of repeated measurements taken at different times *x*_*ij*_. Because observations within the same individuals are dependent, the between and within individual variance should be considered separately. This can be achieved through the LMM.^1^

$$y_{\mathrm{ij}}=\beta_{0i}+\beta_{1i}x_{\mathrm{ij}}+\epsilon_{\mathrm{ij}}$$

where *β*_0*i*_ = *β*_0_ + *b*_0*i*_ and *β*_1*i*_ = *β*_1_ + *b*_1*i*_ are individual intercept and slope terms, combining the population intercept and slope parameters *β*_0_ and *β*_1_ with individual level residuals *b*_0*i*_ and *b*_1*i*_. The residuals are not estimated directly; instead, the variance and covariance of these random effects are estimated. In our study, variance and covariance components are all estimated by using restricted maximum likelihood. Details of estimation of random effects through restricted maximum likelihood are described in detail elsewhere.^3^, ^34^

The LMM assumes a linear relationship between *x* and *y*, which implies that the velocity estimates *β*_1*i*_ are constant and the acceleration is 0. In this section, we introduce approaches which estimate nonlinear subject‐specific trajectories, their velocity, and acceleration.

### 2.1 Polynomial mixed models

Polynomial regression can be extended to the mixed model framework by allowing for random effects *b*_*pi*_ for some or all polynomial terms *x*^*p*^ with *p* = 0, …, *P*

(1) $$y_{\mathrm{ij}}=\sum_{p=0}^{P}\left(\beta_{p}+b_{\mathrm{pi}}\right)x^{p}+\epsilon_{\mathrm{ij}}$$

$$\epsilon_{\mathrm{ij}}∼N\left(0,\sigma_{\epsilon }^{2}\right),b_{\mathrm{pi}}∼N\left(0,\Sigma \right)$$

$$\Sigma =\left(\begin{array}{ccccc} \sigma_{b0}^{2} & \sigma_{b01} & . & . & \sigma_{b0P}\\ \sigma_{b01} & \sigma_{b1}^{2} & . & . & .\\ . & . & . & . & .\\ . & . & . & . & .\\ \sigma_{b0P} & . & . & . & \sigma_{\mathrm{bP}}^{2} \end{array}\right)$$

Individual trajectories for the *d*th derivative (eg, velocity, *d* = 1) can be obtained as follows by using the estimated best linear unbiased predictors (BLUPs)

$${\hat{y}}_{\mathrm{ij}}^{\left(d\right)}=\sum_{p=d}^{P}\frac{p!}{\left(p-d\right)!}\left({\hat{\beta }}_{p}+{\hat{b}}_{\mathrm{pi}}\right)x^{p-d}$$

with at least *P* = 4 (ie, a quartic polynomial) being recommended to achieve smooth estimates of acceleration trajectories. To obtain confidence bands for individual trajectories, we can use the standard matrix notation

Y=Xβ+Zu+ϵ

$$Y=\left[\begin{array}{c} y_{1}\\ \vdots \\ y_{n} \end{array}\right],y_{i}=\left(y_{i1},\ldots ,y_{in_{i}}\right).$$

$$X=\left[\begin{array}{c} X_{1}\\ \vdots \\ X_{n} \end{array}\right],X_{i}=\left[\begin{array}{cccc} 1 & x_{i1} & \ldots & x_{i1}^{P}\\ \vdots & \vdots & \ldots & \vdots \\ 1 & x_{in_{i}} & \ldots & x_{in_{i}}^{P} \end{array}\right],\beta ={\left[\beta_{0}\;\ldots \;\beta_{P}\right]}^{T}$$

$$Z=\left[\begin{array}{cccc} Z_{1} & 0 & \ldots & 0\\ 0 & Z_{2} & 0 & 0\\ 0 & 0 & Z_{3} & 0\\ \vdots & \vdots & \ldots & \vdots \\ 0 & 0 & \ldots & Z_{n} \end{array}\right],Z_{i}=\left[\begin{array}{cccc} 1 & x_{i1} & \ldots & x_{i1}^{P}\\ \vdots & \vdots & \ldots & \vdots \\ 1 & x_{in_{i}} & \ldots & x_{in_{i}}^{P} \end{array}\right]$$

$$u=\left[u_{1}\ldots u_{n}\right],u_{i}={\left[\begin{array}{ccc} b_{0i} & \ldots & b_{\mathrm{Pi}} \end{array}\right]}^{T}$$

$$\hat{y}=\text{BLUP}\left(y\right)={\hat{\sigma }}_{\epsilon }^{2}C{\left(C^{T}C+\hat{B}\right)}^{-1}C^{T}y$$

$$\hat{B}=\left(\begin{array}{cc} 0 & 0\\ 0 & {\hat{\Sigma }}^{-1} \end{array}\right)$$

where $\hat{\Sigma }$ is defined above; **β** and **u** are the vectors of fixed and random effect coefficients, respectively, and **C** is the joint design matrix for the fixed (**X**) and random (**Z**) design matrices, which for polynomial mixed models contains fixed and random terms for each $x_{\mathrm{ij}}^{P}$:

$$C=\left[\begin{array}{cc} X & Z \end{array}\right]$$

The design matrices **X** and **Z** are identical here such that any fixed effect term has a corresponding random effect. The variance of (1) has been derived^34^ as follows:

$$\mathrm{var}\left({\hat{y}}_{\mathrm{ij}}\right)={\hat{\sigma }}_{\epsilon }^{2}C{\left(C^{T}C+\hat{B}\right)}^{-1}C^{T}$$

Therefore, the standard error of individual velocity estimates can be obtained from

$$\mathrm{var}\left({\hat{y}}_{\mathrm{ij}}^{\left(1\right)}\right)={\hat{\sigma }}_{\epsilon }^{2}C^{\left(1\right)}{\left(C^{T}C+\hat{B}\right)}^{-1}{C^{\left(1\right)}}^{T}$$

with **C**^(1)^ being the velocity design matrix, obtained by taking the derivative of the fixed and random design matrices

$$C^{\left(1\right)}=\left[\begin{array}{cc} X^{\left(1\right)} & Z^{\left(1\right)} \end{array}\right]=\left[0\;1\;2x\ldots {\mathrm{Px}}^{P-1}\;0\;1\;2x\ldots {\mathrm{Px}}^{P-1}\;\right]$$

Similarly, we can derive the standard error of individual acceleration estimates from

$$\mathrm{var}\left({\hat{y}}_{\mathrm{ij}}^{\left(2\right)}\right)={\hat{\sigma }}_{\epsilon }^{2}C^{\left(2\right)}{\left(C^{T}C+\hat{B}\right)}^{-1}{C^{\left(2\right)}}^{T}$$

with

$$C^{\left(2\right)}=\left[0\;0\;2\ldots P\left(P-1\right)x^{P-2}\;0\;0\;2\ldots P\left(P-1\right)x^{P-2}\;\right]$$

### 2.2 Spline mixed models

The model proposed above might not be flexible enough if the shapes of the individual curves are complex. Moreover, using a global polynomial basis means that local changes in the data have global effects. Local modeling approaches such as splines do not suffer from this issue. A spline framework is given in the method described by Durban^3^ in which individual curves are modeled as the sum of a population curve and subject‐specific departures from the mean, modeled as penalized splines with random coefficients:

yij=fxij+gixij+ϵij

Both the mean function (*f*) and the individual trajectories (*g*_*i*_) are estimated by using truncated polynomials (which break at predefined knots *κ*_*k*_, *k* = 1, …, *K*) and have their own set of random effect coefficients (*u*_*k*_ and *v*_*ki*_, respectively) that allow for these individual trajectories to be obtained. The *u*_*k*_ allow for a flexible mean trend estimate and are the same for each individual; the *v*_*ki*_ allow for this flexible mean estimate to vary between individuals.

(2) $$y_{\mathrm{ij}}=\sum_{p=0}^{P}\left(\beta_{p}+b_{\mathrm{pi}}\right)x_{\mathrm{ij}}^{P}+\sum_{k=1}^{K}\;u_{k}{{\left(x_{\mathrm{ij}}-\kappa_{k}\right)}_{+}}^{P}+\sum_{k=1^{*}}^{K^{*}}\;v_{\mathrm{ki}}{{\left(x_{\mathrm{ij}}-\kappa_{k^{*}}\right)}_{+}}^{P}+\epsilon_{\mathrm{ij}}$$

where *a*_+_ = *a* if *a* > 0 or 0 otherwise, *b*_*pi*_ ∼ *N*(0, **Σ**), $u_{k}∼N\left(0,\sigma_{u}^{2}\right)$, and $v_{\mathrm{ki}}∼N\left(0,\sigma_{v}^{2}\right)$ with the variance covariance matrix

$$G+\left(\begin{array}{ccc} \sigma_{\mu }^{2}I & 0 & 0\\ 0 & \Sigma & 0\\ 0 & 0 & \sigma_{v}^{2}I \end{array}\right)$$

The use of spline mixed models can lead to an incorrect population mean estimate and incorrect pointwise standard errors,^24^ due to the effect of covariance structure of the individual effects on the population mean estimate. Heckman^25^ pointed out that this could be remedied by ensuring that the function space for the individual effects is a subspace of the function space for the population mean curve. We have used a simple approach to apply this condition which is to select the knots for the individual curves ( $\kappa_{k^{*}})$ as a subset of the knots of the population curves (*κ*_*k*_). As discussed in Durban et al,^3^ *K* should be large enough to ensure the flexibility of the curve, with the knots chosen as quantiles of *x*_*ij*_ with probabilities 1/(*K* + 1), … *K*/(*K* + 1). A truncated spline basis is chosen for their simple mathematical form when formulating derivatives. B‐splines' other basis functions (with more tractable numerical properties) could also be used and are easily implemented in the software and code provided here.

In Durban,^23^ individual trajectories were estimated. Here, we use the derivative estimation of spline mixed models to obtain individual velocity and acceleration trajectories. To achieve smooth estimates of acceleration trajectories, we choose *P* = 4; ie, a quartic truncated spline basis is recommended:

$${\hat{y}}_{\mathrm{ij}}^{\left(d\right)}=\sum_{p=d}^{P}\frac{p!}{\left(p-d\right)!}\;\left({\hat{\beta }}_{p}+{\hat{b}}_{\mathrm{pi}}\right)x^{p-d}+\sum_{k=1}^{K}\frac{P!}{\left(P-d\right)!}\;{\hat{u}}_{k}{{\left(x_{\mathrm{ij}}-\kappa_{k}\right)}_{+}}^{P-d}+\sum_{k=1^{*}}^{K^{*}}\frac{P!}{\left(P-d\right)!}\;{\hat{v}}_{\mathrm{ki}}\;{{\left(x_{\mathrm{ij}}-\kappa_{k^{*}}\right)}_{+}}^{P-d}$$

To obtain confidence bands for individual trajectories, we can use standard matrix notation

Y=Xβ+Zu+ϵ

$$Y=\left[\begin{array}{c} y_{1}\\ \vdots \\ y_{n} \end{array}\right],y_{i}=\left(y_{i1},\cdots ,y_{{\text{in}}_{i}}\right)$$

$$X=\left[\begin{array}{c} X_{1}\\ \vdots \\ X_{n} \end{array}\right],X_{i}=\left[\begin{array}{c} 1\\ \vdots \\ 1 \end{array}\begin{array}{c} x_{i1}\\ \vdots \\ x_{{\text{in}}_{i}} \end{array}\begin{array}{c} \cdots \\ \cdots \\ \cdots \end{array}\begin{array}{c} x_{i1}^{P}\\ \vdots \\ x_{{\text{in}}_{i}}^{P} \end{array}\right],\beta ={\left[\beta_{0}\cdots \beta_{p}\right]}^{T},$$

$$Z=\left[\begin{array}{cccc} Z_{1} & 0 & \cdots & 0\\ 0 & 0 & 0 & 0\\ 0 & 0 & Z_{3} & 0\\ \vdots & \vdots & \cdots & \vdots \\ 0 & 0 & \cdots & Z_{n} \end{array}\right],$$

$$Z_{i}=\left[\begin{array}{cccccccccc} 1 & x_{i1} & \ldots & x_{i1}^{P} & {{\left(x_{i1}-\kappa_{1}\right)}^{P}}_{+} & \ldots & {{\left(x_{i1}-\kappa_{K}\right)}^{P}}_{+} & {{\left(x_{i1}-\kappa_{1^{*}}\right)}^{P}}_{+} & \ldots & {{\left(x_{i1}-\kappa_{K^{*}}\right)}^{P}}_{+}\\ \vdots & \vdots & \vdots & \vdots & \vdots & \vdots & \vdots & \vdots & \vdots & \vdots \\ 1 & x_{in_{i}} & \ldots & x_{in_{i}}^{P} & {{\left(x_{in_{i}}-\kappa_{1}\right)}^{P}}_{+} & \ldots & {{\left(x_{in_{i}}-\kappa_{K}\right)}^{P}}_{+} & {{\left(x_{in_{i}}-\kappa_{1^{*}}\right)}^{P}}_{+} & \ldots & {{\left(x_{in_{i}}-\kappa_{K^{*}}\right)}^{P}}_{+} \end{array}\right],$$

$$u=\left[u_{1}\ldots u_{n}\right],u={\left[\begin{array}{ccc} b_{0i} & \ldots & b_{\mathrm{Pi}} \end{array};u_{1},\ldots ,u_{K};v_{1^{*}i}\ldots ,v_{K^{*}i}\right]}^{T}$$

$$\hat{y}=\text{BLUP}\left(y\right)=C{\left(C^{T}C+\hat{B}+\frac{{\hat{\sigma }}_{\epsilon }^{2}}{{\hat{\sigma }}_{u}^{2}}D\right)}^{-1}C^{T}y$$

where **C** is the joint design matrix

$$C=\left[\underset{\text{fixed terms}}{\underbrace{1\;x\ldots x^{P}}};\underset{\text{random terms}}{\underbrace{1\;x\ldots x^{P}}};\underset{\text{population spline terms}}{\underbrace{{{\left(x-\kappa_{1}\right)}_{+}}^{P}\ldots {{\left(x-\kappa_{K}\right)}_{+}}^{P}}};\underset{\text{individual spline terms}}{\underbrace{{{\left(x-\kappa_{1^{*}}\right)}_{+}}^{P}\ldots {{\left(x-\kappa_{K^{*}}\right)}_{+}}^{P}}}\right]$$

$$\hat{B}=\left(\begin{array}{cc} 0 & 0\\ 0 & {\hat{\Sigma }}^{-1} \end{array}\right)$$

and **D** is a diagonal matrix with *P* + 1 zeros in the first *P* + 1 diagonal elements

$$D=\mathrm{diag}\;\left(0,\;\ldots ,0,1\;\ldots \;,1\right)$$

The variance of (2) is

$$\mathrm{var}\left({\hat{y}}_{\mathrm{ij}}\right)={\hat{\sigma }}_{\epsilon }^{2}C\;{\left(C^{T}C+\hat{B}+\frac{{\hat{\sigma }}_{\epsilon }^{2}}{{\hat{\sigma }}_{u}^{2}}D\right)}^{-1}C^{T}$$

The variance of individual velocity can be found by using

$$\mathrm{var}\left({\hat{y}}_{\mathrm{ij}}^{\left(1\right)}\right)={\hat{\sigma }}_{\epsilon }^{2}C^{\left(1\right)}{\left(C^{T}C+\hat{B}+\frac{{\hat{\sigma }}_{\epsilon }^{2}}{{\hat{\sigma }}_{u}^{2}}D\right)}^{-1}{C^{\left(1\right)}}^{T}$$

where

$$C^{\left(1\right)}=\left[\underset{\text{fixed terms}}{\underbrace{0\;1\;2x\ldots {\mathrm{Px}}^{P-1}\;}};\underset{\text{random terms}}{\underbrace{0\;1\;2x\ldots {\mathrm{Px}}^{P-1}}};\underset{\text{population spline terms}}{\underbrace{P{\left(x-\kappa_{1}\right)}^{P-1}\ldots P{\left(x-\kappa_{K}\right)}^{P-1}}};\underset{\text{individual spline terms}}{\underbrace{P{\left(x-\kappa_{1^{*}}\right)}^{P-1}\ldots P{\left(x-\kappa_{K^{*}}\right)}^{P-1}}}\right]$$

Similarly, the variance of an acceleration trajectory can be obtained through

$$\mathrm{var}\left({\hat{y}}_{\mathrm{ij}}^{\left(2\right)}\right)={\hat{\sigma }}_{\epsilon }^{2}C^{\left(2\right)}{\left(C^{T}C+\hat{B}+\frac{{\hat{\sigma }}_{\epsilon }^{2}}{{\hat{\sigma }}_{u}^{2}}D\right)}^{-1}{C^{\left(2\right)}}^{T}$$

where

$$C^{\left(2\right)}=\left[\underset{\text{fixed terms}}{\underbrace{0\;0\;2\ldots P\left(P-1\right)x^{P-2}}};\underset{\text{random terms}}{\underbrace{0\;0\;2\ldots P\left(P-1\right)x^{P-2}}};\underset{\text{population spline terms}}{\underbrace{P\left(P-1\right){{\left(x-\kappa_{1}\right)}_{+}}^{P-2}\ldots P\left(P-1\right){{\left(x-\kappa_{K}\right)}_{+}}^{P-2}}};\underset{\text{individual spline terms}}{\underbrace{P\left(P-1\right){{\left(x-\kappa_{1^{*}}\right)}_{+}}^{P-2}\ldots P\left(P-1\right){{\left(x-\kappa_{K^{*}}\right)}_{+}}^{P-2}}}\right]$$

### 2.3 Principal component analysis through conditional expectation

Principal component analysis through conditional expectation^7^, ^18^ was developed to estimate individual trajectories in sparse longitudinal data. It is based on functional principal component (FPC) analysis^5^ but adapted to handle irregular and sparse repeated measure data which are common in biomedical applications.

In standard functional PCA, the observations are seen as discrete representations of a collection of underlying smooth functions *f*. The data have a mean function μ(*t*) = *E*[*f*(*t*)] and covariance function *G*(*s*, *t*) =  cov [*f*(*t*), *f*(*s*)] = *E*[(*f*(*t*) − μ(*t*))(*f*(*s*) − μ(*s*))] for some time points *s* and *t* on an interval *T* which is closed and bounded. The first step is to smooth (using local linear smoothers) the pooled data to obtain an estimate of μ. Second, the covariance surface *G*(*s*, *t*) is smoothed, and this fitted surface is then decomposed into an orthogonal expansion in eigenfunctions *ϕ*_*k*_ and nonincreasing eigenvalues *λ*_*k*_

$$G\left(s,t\right)=\sum_{k}\;\lambda_{k}\phi_{k}\left(s\right)\phi_{k}\left(t\right)$$

up to some threshold of variance such that *k* = 1, 2, 3, … FPCs are chosen. The truncation lag was chosen for this study as the minimum number of components required to explain 95% of the variability in the observed data.

Using the Karhunen‐Loève expansion, each individual curve can be represented as

$$f\left(t\right)=\mu \left(t\right)+\sum_{k}\xi_{k}\phi_{k}\left(t\right)$$

where the *ξ*_*k*_ are uncorrelated random variables with mean zero and variance *λ*_*k*_. The *k*th FPC *ϕ*_*k*_ describes the *k*th pattern of variation of the response. This vector is scaled by using the “factor loadings” or FPC scores *ξ*_*k*_ which quantify the correlation between the individual's trajectory and the corresponding FPC. Moving to the case of sparse irregular measurement times *x*_*ij*_, uncorrelated *iid* within subject residuals *ϵ*_*ij*_ are assumed with mean zero and constant variance *σ*^2^. The *ϵ*_*ij*_ are assumed to be independent of the random variables *ξ*_*jk*_. Then for a response *y*_*ij*_

$$y_{\mathrm{ij}}=f\left(x_{\mathrm{ij}}\right)+\epsilon_{\mathrm{ij}}=\mu \left(x_{\mathrm{ij}}\right)+\sum_{k}\xi_{\mathrm{jk}}\phi_{k}\left(x_{\mathrm{ij}}\right)+\epsilon_{\mathrm{ij}}$$

The *ξ*_*jk*_ allow each subject their own estimated smooth trajectory and can be thought of as the individual level random effects *b*_*j*_ encountered in the spline and polynomial mixed models. Pointwise confidence bands can be added to PACE fitted trajectories as derived in Yao,^7^ using

$${\hat{y}}_{\mathrm{ij}}\pm 1.96\sqrt{{\hat{\phi }}_{k}^{T}{\hat{\Omega }}_{k}{\hat{\phi }}_{k}}$$

where ${\hat{\phi }}_{k}={\hat{\phi }}_{k}\left(x_{\mathrm{ij}}\right)$, ${\hat{\Omega }}_{k}=\hat{\Lambda }-\hat{H}{\hat{\Sigma }}_{Y_{i}}^{-1}{\hat{H}}^{T}$, with $\hat{\Lambda }=\mathrm{diag}\left({\hat{\lambda }}_{1},\ldots ,{\hat{\lambda }}_{K}\right)$, $\hat{H}={\left({\hat{\lambda }}_{1}{\hat{\phi }}_{i1},\ldots ,{\hat{\lambda }}_{K}{\hat{\phi }}_{\mathrm{iK}}\right)}^{T}$and ${\hat{\Sigma }}_{Y_{i}}$ is an *n*_*i*_ × *n*_*i*_ matrix with (*j*, *l*) entry ${\left({\hat{\Sigma }}_{Y_{i}}\right)}_{j,l}=\hat{G}\left(x_{\mathrm{ij}},x_{\mathrm{il}}\right)+{\hat{\sigma }}^{2}\delta_{\mathrm{jl}}$, where *δ*_*jl*_ = 1 if *j* = *l* and 0 if *j* ≠ *l*. Derivation of the velocity (*d* = 1) and acceleration (*d* = 2) using the PACE model can be found in Liu^3^:

$${\hat{f}}^{\left(d\right)}\left(x_{\mathrm{ij}}\right)={\hat{\mu }}^{\left(d\right)}\left(x_{\mathrm{ij}}\right)+\sum_{k}{\hat{\xi }}_{\mathrm{jk}}{\hat{\phi }}_{k}^{\left(d\right)}$$

with 95% pointwise confidence bands for velocity and acceleration trajectories obtained by using

$${\hat{f}}^{\left(d\right)}\left(x_{\mathrm{ij}}\right)\pm 1.96\sqrt{{\hat{\phi }}_{k}^{{\left(d\right)}^{T}}\;{\hat{\Omega }}_{k}^{\left(d\right)}{\hat{\phi }}_{k}^{\left(d\right)}\;}$$

where ${\hat{\Omega }}_{k}^{\left(d\right)}=\hat{\Lambda }-{\hat{H}}^{\left(d\right)}{\hat{\Sigma }}_{Y_{i}}^{-1}{\hat{H}}^{{\left(d\right)}^{T}}$. Principal component analysis through conditional expectation is fitted in MATLAB^35^ by using code uploaded by the authors. Full details of PACE and FPCDer are located at http://anson.ucdavis.edu/PACE. We fitted PACE models to simulated and real data by using software developed for MATLAB.

## 3 SIMULATION STUDY

### 3.1 Data

To compare performance of methods for derivative estimation, we simulated data from 2 functions with known velocities and accelerations that mimic standard biomedical applications (Table 1). *f*_1_ has a sharp rise to a well‐defined maximum, followed by slowing decline (eg, the BMI trajectory between birth and age 5 and CD4 trajectories in HIV patients undergoing treatment). *f*_2_ declines throughout, but with a change‐point at which the rate of decline changes (eg, cognitive function in older age). Using these functions, we can assess goodness of fit to the individual observations achieved by each method and crucially, to the true individual velocity and acceleration trajectories.

**Table 1:** Functions used for simulation of velocity and acceleration trajectories

Function	Functional Form	Distributions
***f***_**1**_	$a_{i}+\frac{1}{\frac{c_{i}x}{5}+2b_{i}e^{-16x^{2}}}$	*x* ∈ [0, 1] *a* ~ *N* (0, 1) *b* ~ *N* (0.5,0.14) *c* ~ *N* (3.5,1) *ρ*_*ab*_ = *ρ*_*ac*_ = *ρ*_*bc*_ = 0.2
***f***_**2**_	$a_{i}-b_{i}\cos\left(\frac{5xc_{i}}{4}\left[2x-\pi \right]\right)+6e^{-16x^{2}}$	

**Figure 1:**
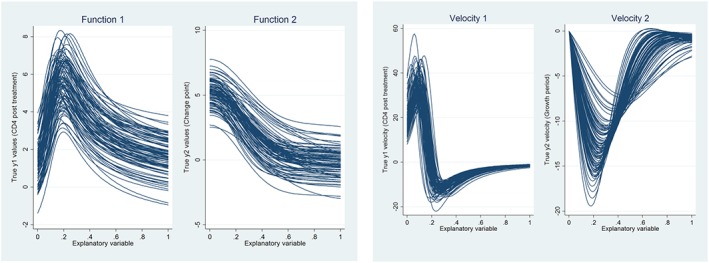
Simulated data and velocity trajectories [Colour figure can be viewed at http://wileyonlinelibrary.com]

We simulated 1000 datasets (code provided in Supplementary file), and within each dataset, individual‐level coefficients were drawn from a multivariate normal distribution such that a set of true individual trajectories *f* (*x*_*ij*_) were generated for each function (eg, Figure 1), where *i* = 1, …, *n* indexes the n individuals in the sample, *j* = 1, …, *n*_*i*_ indexes the *n*_*i*_ observations for individual *i* and *x*_*ij*_ ∈ [0, 1]. Residuals were added to these to generate observations from each function *y*_*ij*_ = *f* (*x*_*ij*_) + *ϵ*, with *ϵ*~*N*(0, *σ*_*ϵ*_) independent and identically distributed.

To compare methods over a variety of potential scenarios, we varied measurement error (*σ*_*ϵ*_ = 0.1, 0.25, 0.5), sample size (*n* = 50, 250, and 1000), and number of measures per individual (*n*_*i*_ = 5, 10, 20). Another important factor which can impact goodness of fit is whether measurement occasions are the same (regular) or different (irregular) between individuals. We simulated an irregular design with roughly 5, 10, and 20 measurements per individual. To simulate an irregular design of around 5 repeated measures, for each individual, we drew a value from a normal distribution with mean 5 and standard deviation 1 and rounded to the nearest integer to give the number of repeated measures for that individual. We then randomly selected the designated number of measurement occasions to include in the simulated dataset for that individual.

The primary setting for analysis was *σ*_*ϵ*_ = 0.25, *n* = 250, and *n*_*i*_ ≈ 10 irregular measures. We report in depth on this simulation and then compare the effect of changing simulation scenarios. Because the functions are known, the true velocity *f*^ (1)^(*x*_*ij*_) and acceleration *f*^ (2)^(*x*_*ij*_) can be obtained for each individual (Figure 1). Using each method under investigation, we can obtain estimated velocity and acceleration trajectories and compare performance through

$$\;\text{bias}=|\;f^{\left(d\right)}\left(x_{\mathrm{ij}}\right)-{\hat{f}}^{\left(d\right)}\left(x_{\mathrm{ij}}\right)|$$

where *d* = 0, 1, 2 represent the bias of the fit, velocity, and acceleration estimates. We present a scaled bias, which is derived by dividing the bias by the standard deviation of the true response and derivatives *f* (*x*_*ij*_), *f*^ (1)^(*x*_*ij*_), and *f*^ (2)^(*x*_*ij*_). This scaled bias allows us to compare the relative bias between curves fitted to the data and derivatives. We compare models for the data, velocity, and acceleration to investigate whether the optimal model for fitting the observed data is always optimal for derivative estimation.

The pointwise coverage of the true trajectories is determined by assessing whether the true curve *f*^ (*d*)^(*x*_*ij*_) is contained in the estimated pointwise interval ${\hat{f}}^{\left(d\right)}\left(x_{\mathrm{ij}}\right)\pm 1.96\times \mathrm{se}\left({\hat{f}}^{\left(d\right)}\left(x_{\mathrm{ij}}\right)\right)$ for all individuals *i* = 1, …, *n*, measurements *j* = 1, …, *n*_*i*_, and derivatives *d* = 0, 1, 2.

### 3.2 Results

The results of the simulation study are summarized in Table 2 for *n* = 250, *n*_*i*_ ≈ 10, and measurement error $\sigma_{\epsilon }^{2}=0.25$. For *f*_1_, the spline mixed model and PACE had lower mean bias (0.22) than the polynomial mixed model (0.30) when fitting trajectories to individuals' repeated data. In estimating velocity and acceleration trajectories, the spline method had lower mean bias (2.2 and 50.6 for velocity and acceleration, respectively) than both PACE (3.3 and 74.3) and the polynomial approach (4.9 and 104.0). Bias increased when fitting velocity and acceleration trajectories (spline mean scaled bias 18% and 34%, respectively), compared with fitting trajectories to data (13%).

**Table 2:** Results comparing the bias (true—fitted value) of 3 methods across 2 functions in 1000 simulations of *n* = 250 individuals, measured approximately 10 times each, with measurement error of N(0,0.25) added

Function	Derivative	Method	Mean Bias (SD)	Bias (%)	Coverage (%)
***f***_**1**_	Fit	PACE	0.22 (0.02)	13	88.9
		PMM	0.30 (0.01)	18	99.1
		SPMM	0.22 (0.02)	13	99.2
	Velocity	PACE	3.32 (0.59)	26	57.0
		PMM	4.89 (0.09)	39	58.5
		SPMM	2.22 (0.60)	18	87.1
	Acceleration	PACE	74.34 (13.04)	51	41.2
		PMM	104.04 (1.57)	71	28.8
		SPMM	50.63 (12.45)	34	78.0
***f***_**2**_	Fit	PACE	0.18 (0.02)	9	85.4
		PMM	0.17 (0.01)	8	99.6
		SPMM	0.17 (0.01)	8	99.9
	Velocity	PACE	2.34 (0.55)	50	46.3
		PMM	1.52 (0.05)	32	76.6
		SPMM	1.30 (0.22)	28	89.7
	Acceleration	PACE	37.04 (12.06)	101	37.0
		PMM	17.35 (0.55)	47	25.0
		SPMM	13.67 (3.59)	37	78.3

All 3 methods had similar mean bias (0.17‐0.18) when fitting curves to the *f*_2_ data. The spline mixed model had the lowest mean bias when estimating velocity (1.3) and acceleration (13.7) trajectories for *f*_2_. Once again, the bias increased when estimating velocity (spline mean scaled bias 28%) and acceleration (37%) compared with estimating trajectories of *f*_2_ (8%).

**Figure 2:**
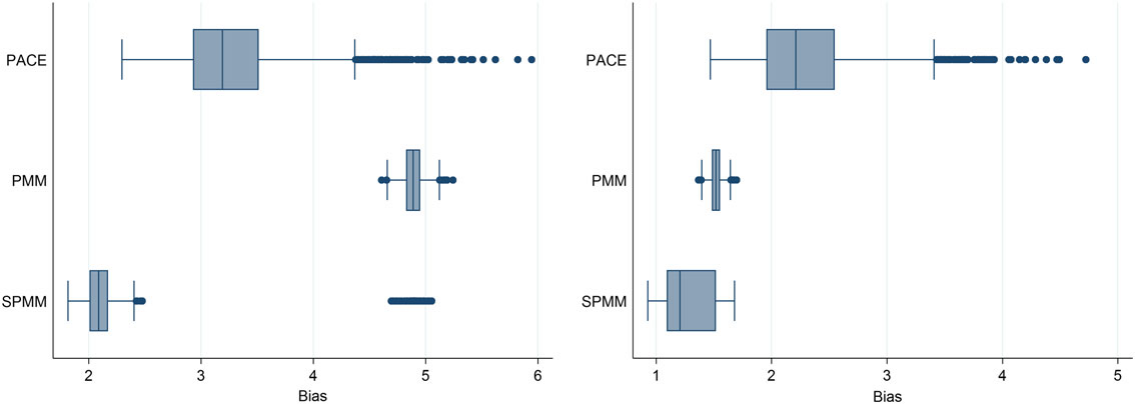
Bias in estimating velocity trajectories of ***f***_**1**_ (left) and ***f***_**2**_ (right) [Colour figure can be viewed at http://wileyonlinelibrary.com]

In consistency between simulations, the PACE method showed large fluctuations between low and high biases (Figure 2). Both mixed model methods shared consistency between simulations, with some outliers observed for the spline approach for *f*_1_.

#### 3.2.1 Coverage

Table 2 also presents the pointwise confidence bands. Confidence bands from both mixed model methods almost always contained the true underlying individual data (coverage ≈ 99% for *f*_1_ and *f*_2_), while PACE confidence bands did not achieve nominal coverage of individual data (89% *f*_1_ and 85% *f*_2_). Coverage decreased when estimating velocity and acceleration, with no method achieving nominal coverage. The spline mixed model had the highest coverage of true velocity (87% and 90% for *f*_1_ and *f*_2_, respectively) and acceleration (78% for both functions). The polynomial mixed model had poor coverage of the true velocity (59% and 77% for *f*_1_ and *f*_2_, respectively), and this deteriorated for acceleration (29% and 36% for *f*_1_ and *f*_2_, respectively). Pointwise confidence bands for PACE derivative estimates did not achieve nominal coverage for velocity (57% and 46% for *f*_1_ and *f*_2_, respectively) or acceleration (41% and 37% for *f*_1_ and *f*_2_, respectively).

#### 3.2.2 Changing experimental design

Table 3 summarizes the bias in estimation of velocity when the experimental design is changed (measurement error, sample size, number of measures per individual, and regular/irregular times). Increasing measurement error had minimal effect on the polynomial method for estimating velocity trajectories, with some reduction in bias for both the spline and PACE methods. Principal component analysis through conditional expectation was sensitive to sample size, with bias dropping from 4.3 to 2.3 for *f*_1_ and 3.0 to 1.6 for *f*_2_ when the sample size increased from 50 to 1000. Sample size had a similar effect on velocity estimates for the spline mixed model, whereas the polynomial mixed model appeared robust to sample size changes.

**Table 3:** Mean bias in velocity between the 3 methods under different scenarios

Function	Method	Mean Bias (SD)		
Changing measurement error		$\sigma_{\epsilon }^{2}=0.5$	$\sigma_{\epsilon }^{2}=0.25$	$\sigma_{\epsilon }^{2}=0.1$
***f***_**1**_	PACE	3.47 (0.60)	3.32 (0.59)	3.28 (0.58)
	PMM	4.89 (0.11)	4.89 (0.09)	4.9 (0.09)
	SPMM	2.19 (0.32)	2.22 (0.60)	2.10 (0.31)
***f***_**2**_	PACE	2.49 (0.59)	2.34 (0.55)	2.31 (0.56)
	PMM	1.53 (0.06)	1.52 (0.05)	1.47 (0.05)
	SPMM	1.44 (0.19)	1.30 (0.22)	1.17 (0.21)
Changing sample size		*n* = 50	*n* = 250	*n* = 1000
***f***_**1**_	PACE	4.34 (0.76)	3.32 (0.59)	2.34 (0.28)
	PMM	4.89 (0.21)	4.89 (0.09)	4.89 (0.05)
	SPMM	4.66 (0.76)	2.22 (0.60)	2.05 (0.05)
***f***_**2**_	PACE	3.01 (0.68)	2.34 (0.55)	1.55 (0.23)
	PMM	1.53 (0.11)	1.52 (0.05)	1.52 (0.03)
	SPMM	1.53 (0.11)	1.30 (0.22)	1.08 (0.04)
Changing frequency of measurement (irregular design scenario)		*n*_*i*_ = 5	*n*_*i*_ = 10	*n*_*i*_ = 20
***f***_**1**_	PACE	4.19 (0.66)	3.32 (0.59)	2.71 (0.42)
	PMM	4.91 (0.11)	4.89 (0.09)	4.89 (0.08)
	SPMM	4.84 (0.08)	2.46 (0.86)	2.01 (0.08)
***f***_**2**_	PACE	3.11 (0.68)	2.34 (0.55)	1.85 (0.35)
	PMM	1.54 (0.06)	1.52 (0.05)	1.46 (0.04)
	SPMM	1.51 (0.13)	1.30 (0.22)	1.06 (0.09)
Changing frequency of measurement (regular design scenario)		*n*_*i*_ = 5	*n*_*i*_ = 10	*n*_*i*_ = 20
***f***_**1**_	PACE	4.86 (0.07)	4.56 (0.08)	1.97 (0.08)
	PMM	4.84 (0.08)	4.46 (0.07)	4.57 (0.07)
	SPMM	4.84 (0.08)	4.15 (0.5)	3.56 (0.69)
***f***_**2**_	PACE	2.15 (0.04)	1.55 (0.04)	1.20 (0.05)
	PMM	1.47 (0.04)	1.43 (0.04)	1.40 (0.04)
	SPMM	1.47 (0.04)	1.12 (0.15)	1.00 (0.05)

Using an irregular design, the spline and PACE models were affected by increasing frequency of measurement, with a considerable drop in bias evident when measurement frequency was increased from 5 to 10 to 20 per individual. This effect was even stronger when a regular design was used (all individuals had the same measurement times) with bias dropping from 4.9 to 2.0 in *f*_1_ and 2.2 to 1.2 in *f*_2_ by using PACE. The polynomial model was robust to the changes in design, with the only marked improvement observed when moving from an irregular to regular design (bias decreasing from 4.9 to 4.5 for 10 measurements per individual for *f*_1_). The spline method was strongly affected by frequency of measurement in a regular design, where bias decreased from 4.8 to 3.6 in *f*_1_ and 1.5 to 1.0 in *f*_2_ when frequency of measurement increased from 5 to 20 per individual.

**Figure 3:**
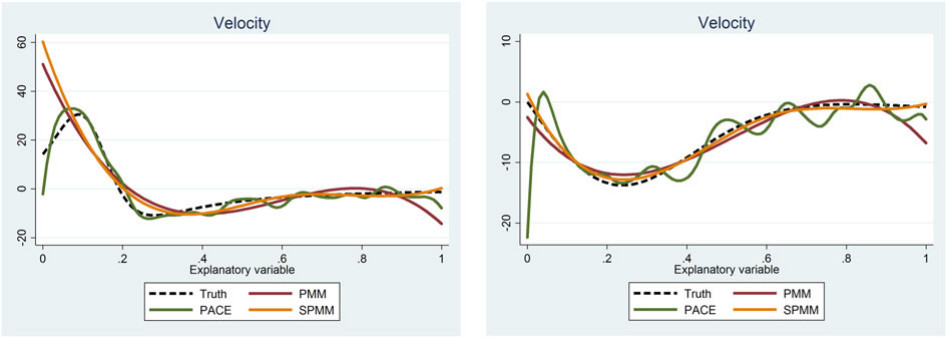
Estimates of a single velocity trajectory simulated from ***f***_**1**_ (left) and ***f***_**2**_ (right), using all 3 methods with the true velocity the black dashed line [Colour figure can be viewed at http://wileyonlinelibrary.com]

**Figure 4:**
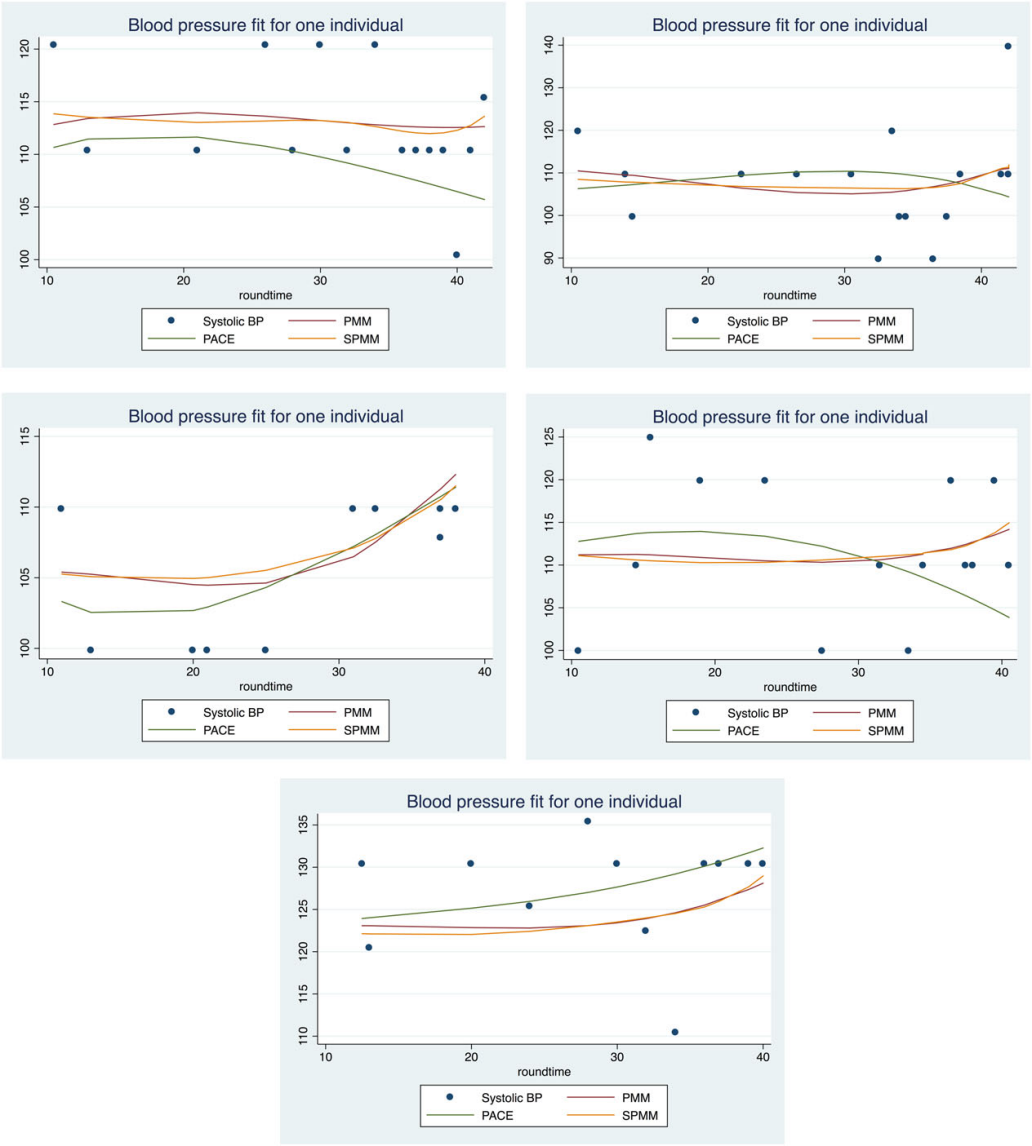
Systolic blood pressure (BP) for 5 individuals with fitted trajectories from the polynomial mixed model (red), spline model (yellow), and principal component analysis through conditional expectation (green). Data have been anonymized to prevent publication of revelatory information: These 5 individuals were selected at random, a random number was added to both blood pressure and fitted values (to leave the relationship undisturbed), a random 75% of BPs are shown, and gestation has been rounded to the nearest week [Colour figure can be viewed at http://wileyonlinelibrary.com]

As an illustration, we have randomly selected 1 velocity trajectory from each of the 2 functions and shown the estimated trajectories using each method (Figure 3). In *f*_1_, PACE was the only method that identified an initial increase in velocity, with some superfluous oscillations when the velocity becomes flat later in the range. The polynomial model has an extra dip toward the end, which is an artifact of the polynomial used (quartic leading to cubic velocity) which must have 2 local minima/maxima. On the other hand, the spline method remains flat in the right tail because it is locally flexible (ie, can change shape in different sections of *x*). The spline model excels in capturing the true velocity of *f*_2_. Once again, we see the polynomial take a superfluous dip at the right tail. Principal component analysis through conditional expectation suggests an early rapid increase in velocity that is not present. This leads to heavy bias when applying the PACE method. Further, there seems to be a general under‐smoothing when using PACE to estimate derivatives of *f*_2_ (Figure 4).

### 3.3 Summary of simulation study

All 3 methods had similar goodness of fit for *f*_1_ and *f*_2_, but the spline approach outperformed the others in derivative estimation. This suggests that a good fit to the data may not always reflect a better fit to the derivative. In more complex scenarios, we should not always trust derivative estimates even where a model fits the observed data well. It is therefore crucial to examine velocity and acceleration estimates. A simple example of this is when using polynomial models; choosing a cubic basis to fit data may achieve a good fit but means that the velocity trajectories will follow a quadratic path. This can lead to poor estimates, often in the tails (Figure 6). Here, we should use a higher order polynomial or preferably a locally flexible method to refit the data to achieve more flexible velocity estimates. Another example is the PACE fit shown in Figure 3, where there is clearly undersmoothing in the individual prediction. In this case, we should refit the data with an increased smoothing parameter.

Empirical testing of derivative estimation can only go so far in finding an optimal approach because simulations are limited by the chosen functional forms. Thus, the choice of which method to use should also include discussion of efficiency, ease of use, and interpretation. Polynomial mixed models are easy to implement in any statistical software. Derivatives and their standard errors can be obtained from the estimated BLUPs, as we have shown. However, a global polynomial means that local changes, for example, due to an observation with high measurement error, will have an effect across the entire estimated trajectory. Further, the degree of the polynomial model must be chosen by the user not just to fit curves to the data but to allow for smooth velocity or acceleration. As the degree of the polynomial increases, so do convergence issues when fitting mixed models. Fractional polynomials^36^, ^37^ offer an alternative to the simple specification given in the current article and allow for more flexible fits to the mean trend and individual trajectories. However, this method still employs a global polynomial basis. The spline approach has many of the benefits of the polynomial model, such as ease of implementation. One drawback is the subjective choice of knots, although we recommend using equidistant knots which allow a flexible mean curve. The PACE method has been implemented in MATLAB, and the data need to be carefully arranged before fitting the model.

## 4 APPLICATION TO LONGITUDINAL BLOOD PRESSURE DATA

### 4.1 Blood pressure in pregnancy data

This study uses systolic (SBP) and diastolic BP (DBP) measurements recorded as part of the Avon Longitudinal Study of Parents and Children (ALSPAC).^38^, ^39^ Avon Longitudinal Study of Parents and Children recruited 14 541 pregnant women with expected delivery dates between April 1991 and December 1992. The study website contains details of all the data that are available through a fully searchable data dictionary (http://www.bris.ac.uk/alspac/researchers/data-access/data-dictionary). Approximately 182 059 measurements of SBP and DBP were available from 13 241 women (median 14 measures per person, range 1‐49) during pregnancy. The study includes 5590 (42%) and 7651 (58%) nulliparous and multiparous women, respectively.

High BP in pregnancy after 20 weeks of gestation is used to diagnose hypertensive disorder of pregnancy (HDP; preeclampsia and gestational hypertension), which are associated with adverse health outcomes for the mother and baby.^40^, ^41^ The trajectory of BP in women across pregnancy is nonlinear with, typically, a decline in early pregnancy to a nadir at around 18 to 20 weeks of gestation, followed by gentle rise that increases in rate in late pregnancy, from around 34 to 36 weeks.^42^, ^43^ Thus, it would be clinically useful to detect those women with BP that decreases little in early pregnancy, rises more rapidly than expected (or than on average for a given population) in late pregnancy, or that starts to rise earlier than expected, and then to monitor these women carefully because they are at higher risk of developing adverse perinatal outcomes related to HDP. This is also useful because clinicians often question the arbitrary nature of the thresholds used to define high BP and because women can cross a threshold from very different trajectories—eg, they can start pregnancy with a higher BP (which does not cross HDP thresholds), decline less in early pregnancy, or start to increase at an earlier time. Previous studies have found evidence that nulliparous women are more at risk of HDP than multiparous women, with this appearing to be reflected in higher BP from early in pregnancy.^44^, ^45^

There were 2 aims to this analysis: (i) to estimate the gestational age at which BP starts to increase for each woman and (ii) whether the effect of parity changes with duration of pregnancy. Aim (i) is an individual estimate, and we report whether evidence for a change in BP was found and the typical starting week of this change region across all women. Aim (ii) is a group comparison of BP change and we report velocity difference curves (typical velocity in multiparous women—typical velocity in nulliparous women) with confidence bands using each method. When the confidence bands contain zero, there is no evidence for a difference in BP change between multi‐ and nulliparous women; when the confidence bands are fully negative, this provides evidence that nulliparous women have faster changing BP at that week of gestation. Models were fitted to nulliparous and multiparous women separately to allow for different pattern of variability over time, rather than combining into a shared random effect matrix or FPC decomposition. To allow for smooth velocity estimates, cubic polynomials and a cubic spline basis were used, with *K* = 5 equidistant knots for the mean curve (ie, at the 0.16, 0.33, 0.5, 0.66, and 0.86 quantiles) and K* = 2 equidistant knots used for the subject specific trajectories (ie, at the 0.33 and 0.66 quantiles). We found that this knot selection led to flexible curves for individual trajectories, with fewer K* < *K* allowing more efficient computation with very little difference in model fit. Truncation lag *K* for the PACE approach was the number of components required to 95% of the variability in the observed BP data.

We also report on the goodness of fit to the observed repeated BP data by using the mean absolute error (MAE) of the fit to the data, ie, $\;|y_{\mathrm{ij}}-\hat{f}\left(x_{\mathrm{ij}}\right)|$ for all *i* and *j*.

### 4.2 Analysis of blood pressure during pregnancy

#### 4.2.1 Comparison of methods

In Table 4 the MAE provides us with a measure of performance in fitting trajectories to the SBP and DBP data. Here, we see the spline approach (MAE 6.89 for SBP and 5.15 for DBP) giving a better fit than the polynomial (MAE 7.04 for SBP and 5.36 for DBP) and PACE models (MAE 8.18 for SBP and 6.71 for DBP).

**Table 4:** Mean absolute error of BP and estimated week of BP increase

Outcome	Method	MAE (SD)	Women With Increase (% of Total)	Mean Week of Increase (SD)
SBP	PACE	8.18 (7.29)	2 364 (18)	26 (9)
	PMM	7.04 (5.75)	18 (0.1)	7 (2)
	SPMM	6.89 (5.64)	10 039 (75)	35 (5)
DBP	PACE	6.71 (6.29)	2 591 (19)	27 (10)
	PMM	5.36 (4.32)	23 (0.2)	22 (15)
	SPMM	5.15 (4.15)	10 851 (81)	35 (5)

#### 4.2.2 Estimating the start of blood pressure increase

Using the polynomial mixed model, we found evidence for regions of increasing BP in very few women in ALSPAC, ie, just 18 (0.1%) and 23 (0.2%) women for SBP and DBP, respectively (Table 4). The spline model found evidence for regions of increasing BP in many more women for both SBP (10 039, 75%) and DBP (10 851, 81%). Almost all of these women began to have increasing SBP (9902, 99%) or DBP (10 706, 99%) after the 20‐week threshold for diagnosis of HDP. Principal component analysis through conditional expectation identified 2364 (18%) and 2591 (19%) of women as having increasing BP in pregnancy, with roughly a third of these beginning to increase in SBP (787, 33%) or DBP (900, 35%) before 20 weeks' gestation. On average, the PACE model estimated that BP increase occurred 8 to 9 weeks earlier compared with the spline model.

#### 4.2.3 The association of parity and blood pressure change

For both SBP and DBP, each model identified a difference in BP velocity, where women with first time pregnancies tended to experience a more positive BP velocity (ie, slower decrease and faster increase) compared with those women who had a previous pregnancy (Figure 6). All models showed wider confidence bands at the boundaries, where there are less data. The major difference in the findings between models is the interaction from early pregnancy to week 15 evident in both the polynomial mixed model and PACE, but not the spline model. Indeed, using PACE a faster BP increase is identified across almost all weeks of gestation.

### 4.3 Summary of blood pressure findings

**Figure 5:**
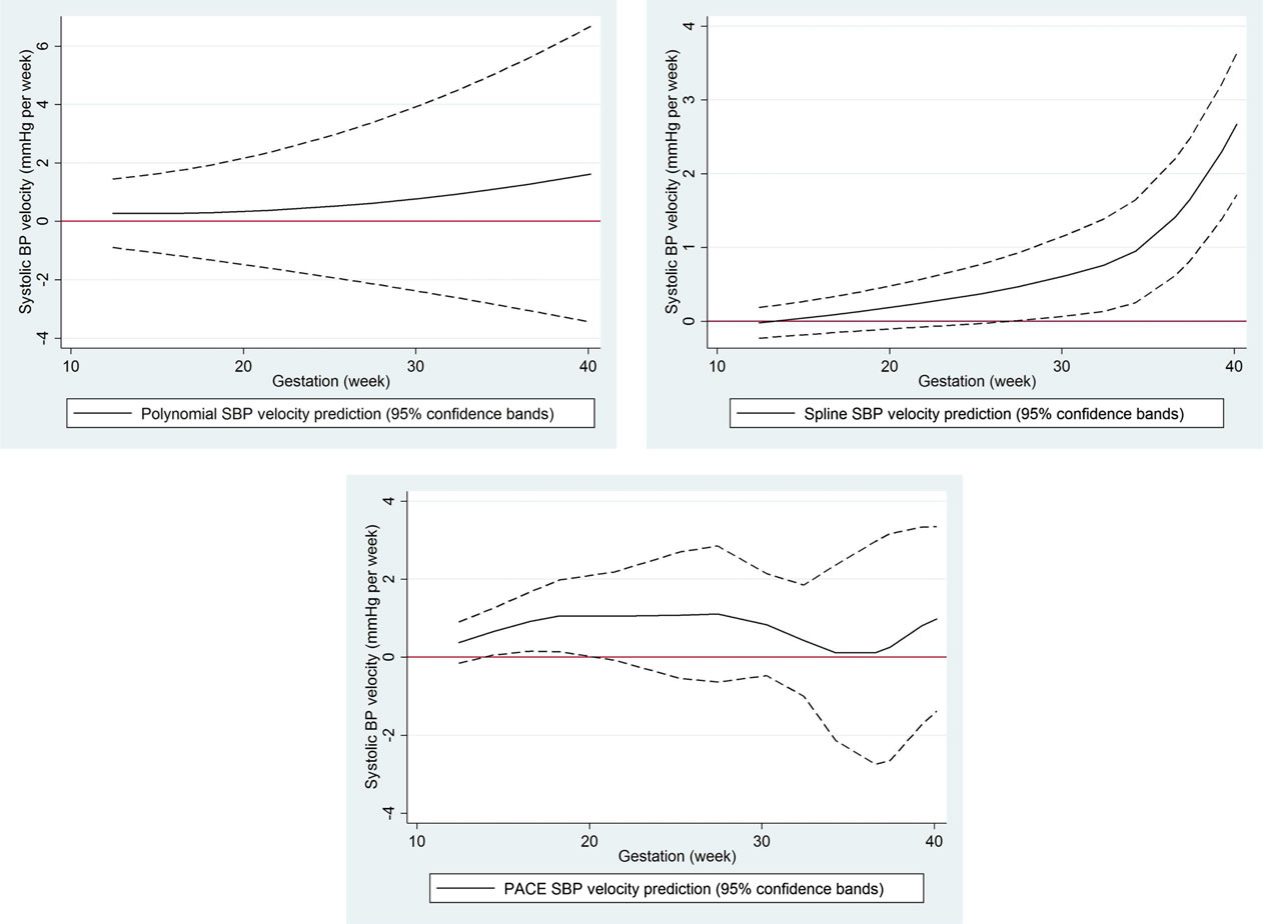
Systolic blood pressure (BP) velocity trajectories with confidence bands for 1 woman during pregnancy estimated by using the polynomial mixed model (top), spline model (middle), and principal component analysis through conditional expectation (bottom). Confidence bands below/above zero give statistical evidence for a decrease/increase in BP at that gestation week [Colour figure can be viewed at http://wileyonlinelibrary.com]

We applied these methods to repeated measures of BP taken during pregnancy, with 2 goals: (i) to test for, and estimate, BP change regions for each woman and (ii) to investigate whether multi‐ and nulliparous women had different change in BP during pregnancy. For both aims, the methods gave vastly different results. The polynomial, spline, and PACE methods found that 0.1%, 75%, and 18% of women had a SBP change region during their pregnancy, with these changes being estimated to begin at an average of 7, 35, and 26 weeks' gestation, respectively. From Figure 5, the lack of evidence using the polynomial model may be due to confidence bands which are too wide, combined with individual predictions which are too smooth. The spline prediction appears more flexible with tighter bands, which allows for a change region to be detected from about week 29 onward for this individual. The PACE prediction appears flexible and with reasonable confidence bands; however, from our simulations, the spline method is likely to have lower bias in estimating velocity trajectories. Unless there is good a priori evidence to assume a polynomial relationship with time, we recommend using the spline mixed effects models, based on our simulation results.

**Figure 6:**
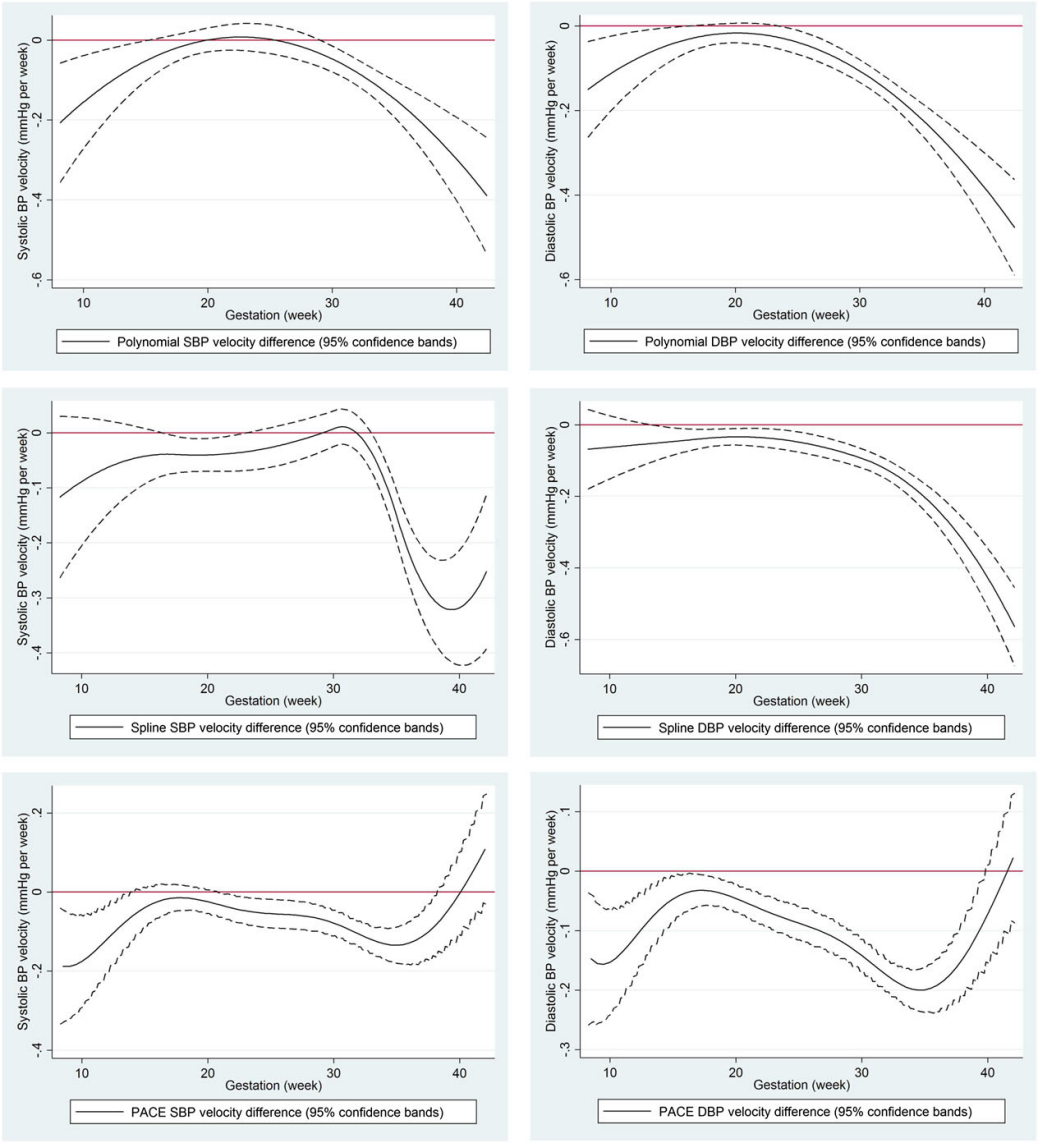
Investigating an interaction between parity and systolic blood pressure change (left column); parity and diastolic blood pressure (right column). The plots show the difference in velocity (previous pregnancy minus first pregnancy) as estimated by the polynomial mixed model (top row), spline mixed model (middle row), and principal component analysis through conditional expectation (bottom row) along with 95% confidence bands. Confidence bands below/above zero give statistical evidence for an interaction at that gestation week [Colour figure can be viewed at http://wileyonlinelibrary.com]

In the second aim of the BP data, we investigated the association of parity with BP change. The difference curves with confidence bands in Figure 6 can be thought of as interaction functions with confidence bands, rather than traditional interaction coefficients with confidence intervals. These plots are much more informative because we can identify different group effects at different study times. All 3 methods identify parity differences in BP change during pregnancy, but at different times. Both PACE and the polynomial methods find that first time mothers have slower declines of SBP and DBP in early pregnancy, up to 13 or 14 weeks' gestation. All 3 methods find evidence a more positive DBP velocity (ie, slower decline in early pregnancy and faster increase in later pregnancy) in nulliparous women for most of their pregnancy. The PACE method estimates that BP change is similar in late pregnancy, while both mixed models find strong evidence for higher BP change in nulliparous women. From our simulations, the spline method is very likely a closer reflection of the truth, ie, finding that there is no difference in BP change in early pregnancy between nulliparous and multiparous mothers.

It is known that women who develop an HDP have a steeper increase in BP during the second half of pregnancy than women who remain normotensive.^40^, ^41^ Given that HDP is defined by a threshold of high BP,^46^ it is also possible that women with a slower BP decline in early pregnancy are more likely to develop HDP. Preeclampsia is the most severe form of HDP, and some studies have suggested that early‐onset preeclampsia (at or before 33 weeks' gestation) is more strongly associated with the risk of adverse outcomes such as stillbirth, preterm birth, and low birth weight than late‐onset preeclampsia.^47^ Thus, the time at which BP begins to show an increase during pregnancy may be a predictor of risk of both HDP and the associated adverse outcomes. The rate of change of BP should be interpreted clinically rather than statistically, with regions of change assessed for clinical importance. The ability to identify whether a woman's BP is stable or on an upward trajectory at any given antenatal appointment would also be useful in making clinical decisions about her future monitoring schedule and help with the early detection of the development of HDP.

Studies of parity and BP in pregnancy have tended to compare the levels of BP in different trimesters between multiparous and nulliparous women, generally finding that BP is higher on average for women in their first pregnancies, particularly in the third trimester.^44^, ^45^ We have focused on differences in the velocity of BP across pregnancy by parity, and our findings suggest that the difference in BP levels between nulliparous and multiparous women in late pregnancy is likely to be partly driven by a greater rate of increase in BP from 30 weeks onward in nulliparous women. This agrees with previous analysis of the ALSPAC data by using linear spline multilevel models,^42^ but the methods we have used here allow for the velocity to vary across gestation and calculation of confidence bands for this velocity at each gestational age, whereas the previous analysis assumed a constant velocity between knot points with only estimation of standard errors for this constant rate of change.

## 5 DISCUSSION

In this paper, we developed derivative estimation for polynomial and penalized spline mixed models. To our knowledge, this paper outlines derivative estimation for longitudinal data analysis in the mixed model framework for the first time. We have derived standard error for these estimates to allow confidence bands and therefore inferences from these derivatives to be made.

The spline derivative estimator performed better than the polynomial mixed model and the established PACE approach. Below nominal coverage for derivative estimation was observed for each method, possibly due to the complexity of the simulations, although the spline mixed model confidence bands performed best with coverage of 90% for velocity and 80% for acceleration. The polynomial and PACE approaches had low coverage of true velocity (46‐77%) and acceleration (29‐41%) trajectories. For the polynomial model, this may be due to poor fit, such that bands have no chance to cover the underlying truth, rather than poor estimation of the standard error—we can see in Table 2 that coverage improves as bias decreases. In practice, data from the underlying velocity are unobserved, so using the polynomial result would give misleading estimates. The poor performance of PACE confidence bands may be due to the extra uncertainties in the FPC decompositions, which have been accounted for with corrected confidence bands in other studies.^29^ The extension of these methods to derivative estimation using PACE would likely increase coverage. Furthermore, because data were generated by using a single function rather than a linear combination of basis functions, the design of our simulation study may have led to the poor performance of PACE (and indeed our spline model). The decision to avoid simulation from basis functions was made because the choice of basis function could then have benefitted either the spline or PACE methods.

The methods were tested comprehensively on 2 functions and across several experimental scenarios. The chosen functions represent common biomedical applications, one with a clearly defined change in direction, another with a more subtle change. Confidence bands were developed and compared between the methods in the simulation. The BP example demonstrated the strengths of the derivative estimation approach in estimating change points and testing for interactions in nonlinear data. However, there were limitations. There was a large discrepancy between results in the BP application. While we have provided a discussion of the reasons underlying these differences, we tested just 2 relatively simple functions. Future studies may wish to carry out simulations tailored to their biomedical application before carrying out derivative estimation.

When investigating change in an outcome, derivative estimation is a useful method. We have developed methods for estimating confidence bands for individual trajectories which can be used to graphically identify change regions. We recommend using spline mixed models for derivative estimation in longitudinal data analysis.

## FINANCIAL SUPPORT

This research was specifically funded by the UK Medical Research Council grant MR/M020894/1. The Wellcome Trust (WT087997MA), US NIH (R01 DK077659), and the British Heart Foundation (SP/07/008/24066) provided funds for the data collected in the ALSPAC mothers that are used in the example of BP change. The UK Medical Research Council and the Wellcome Trust (grant ref: 102215/2/13/2) and the University of Bristol provide core support for ALSPAC. A.J.S., D.A.L., and K.T. all work in a unit that receives support from the University of Bristol and UK Medical Research Council (MC_UU_12013/5 and MC_UU_12013/7). D.A.L. is an NIHR senior investigator (NF‐SI‐0611‐10196). The research of Maria Durban was supported by the Spanish Ministry of Economy and Competitiveness grant MTM2014‐52184.

## ETHICAL APPROVAL

Ethical approval for the study was obtained from the ALSPAC Ethics and Law Committee and the Local Research Ethics Committees.

## DECLARATION OF CONFLICT OF INTEREST

None declared.

## Supporting information

Figure S1. Simulated acceleration trajectories
Figure S2. Bias in estimating acceleration trajectories of *f*_1_ (left) and *f*_2_ (right)
Table S1. Mean bias in acceleration between the three methods under different experimental scenarios

Supporting info item

Supporting info item

Supporting info item
