# Supplementary material for: Derivative estimation for longitudinal data analysis: Examining features of blood pressure measured repeatedly during pregnancy
Source: Stat Med. 2018 May 20;37(19):2836–54. doi: 10.1002/sim.7694 (PMC6099422; doi:10.1002/sim.7694)
Supplement: Supplementary file 1 — Figure S1. Simulated acceleration trajectories Figure S2. Bias in estimating acceleration trajectories of f1 (left) and f2 (right) Table S1. Mean bias in acceleration between the three methods under different experimental scenarios [file SIM-37-2836-s001.docx]

# Supplementary material

# Stata code

- 1. Code to generate simulated data

quietly {

forvalues dat = 1(1)1000 {

clear all

cd "./Simulation study/Data"

set obs 100000

* generate id. 1000 individuals measured 100 times

egen id = seq(), f(1) t(1000) b(100)

label var id "Individual identifier"

* generate x. 100 equally spaced values between 0 and 1

egen x = seq(), f(1) t(100) b(1)

replace x = (x-1)/99

label var x "Explanatory variable"

* generate random vars a, b and c from mvn

* means of 0, 0.5 and 3.75 for a, b and c [found through testing values]

matrix m = (0 \ 0 \ 0)

* sd of 1, 0.14 and 0.7 for a, b and c [trying to keep b between 0.1 and 1, c between 1 and 6.5]

matrix sd = (1 \ 0.14 \ 0.7)

* corr 0.2 between a, b and c [arbitrary]

matrix d = (1, 0.2, 0.2 \ 0.2, 1, 0.2 \ 0.2, 0.2, 1)

* values of b<0.1, b>1, c<1 or c>6.5 blow up some of the functions [e.g. ~1/0]

* starting vals for while loop

gen minb = 0

gen maxb = 10

gen minc = 0

gen maxc = 10

gen re_draw = 1

gen seed = .

label var seed "Seed used to simulate RE"

local re_draw = 1

set more off

qui {

* set the seed for each dataset and RE draw

* (seed+1)^3 used to remove chance of getting same seed twice, e.g. (dat=2, redraw=3) = (dat=3, redraw=2)

local seed = ((`dat'+1)^3)+((`re_draw'+1)^3)

set seed `seed'

* start loop

while (minb < -0.4 | maxb > 0.5 | minc < -2.75 | maxc > 2.75) {

cap drop a b c

drawnorm a b c, corr(d) sds(sd) means(m)

* person level RE

bysort id: replace a = a[1]

bysort id: replace b = b[1]

bysort id: replace c = c[1]

* checking b between 0.1 and 1, c over 0.5

qui su b

replace minb = r(min)

replace maxb = r(max)

qui su c

replace minc = r(min)

replace maxc = r(max)

replace re_draw = re_draw + 1

local re_draw = `re_draw' + 1

replace seed = `seed'

}

}

drop min* max*

replace re_draw = re_draw - 1

label var re_draw "Number of draws to select a, b, c"

label var a "Individual intercept"

label var b "Individual curve shift b"

label var c "Individual curve shift c"

* true values of y1-y5 for all x

cap drop y*

gen y1 = a + 1/((c+3.75) * x/5 + (b+0.5) * 2 * exp(-16 * x))

label var y1 "True y1 values (CD4 post treatment)"

gen y2 = a - cos((x*(c+3.75)/4)*(2*x - (22/7))) + 6*exp(-16*(b+0.5)*x^2)

label var y2 "True y2 values (Change point)"

* true first and second derivatives for each function [found using D() in R, not by hand!]

* f1

gen v1 = -(((c + 3.75)/5 - (b + 0.5) * 2 * (exp(-16 * x) * 16))/((c + 3.75) * ///

x/5 + (b + 0.5) * 2 * exp(-16 * x))^2)

label var v1 "True y1 velocity (CD4 post treatment)"

gen a1 = -((b + 0.5) * 2 * (exp(-16 * x) * 16 * 16)/((c + 3.75) * x/5 + ///

(b + 0.5) * 2 * exp(-16 * x))^2 - ((c + 3.75)/5 - (b + 0.5) * ///

2 * (exp(-16 * x) * 16)) * (2 * (((c + 3.75)/5 - (b + 0.5) * ///

2 * (exp(-16 * x) * 16)) * ((c + 3.75) * x/5 + (b + 0.5) * ///

2 * exp(-16 * x))))/(((c + 3.75) * x/5 + (b + 0.5) * 2 * ///

exp(-16 * x))^2)^2)

label var a1 "True y1 acceleration (CD4 post treatment)"

* f2

gen v4 = sin((x * (c + 3.75)/4) * (2 * x - (22/7))) * ((c + 3.75)/4 * ///

(2 * x - (22/7)) + (x * (c + 3.75)/4) * 2) + 6 * (exp(-16 * ///

(b + 0.5) * x^2) * (-16 * (b + 0.5) * (2 * x)))

label var v4 "True y2 velocity (Change point)"

gen a4 = cos((x * (c + 3.75)/4) * (2 * x - (22/7))) * ((c + 3.75)/4 * ///

(2 * x - (22/7)) + (x * (c + 3.75)/4) * 2) * ((c + 3.75)/4 * ///

(2 * x - (22/7)) + (x * (c + 3.75)/4) * 2) + sin((x * (c + ///

3.75)/4) * (2 * x - (22/7))) * ((c + 3.75)/4 * 2 + (c + 3.75)/4 * ///

2) + 6 * (exp(-16 * (b + 0.5) * x^2) * (-16 * (b + 0.5) * ///

(2 * x)) * (-16 * (b + 0.5) * (2 * x)) + exp(-16 * (b + 0.5) * ///

x^2) * (-16 * (b + 0.5) * 2))

label var a4 "True y2 acceleration (Change point)"

* need to reject data if acceleration starts positive in function 4 [feature of interest is first positive accel]

bysort id: gen a4_ob1 = a4[1]

su a4_ob1

local stopper = r(max)

if `stopper' > 0 {

exit

}

drop a4_ob1

* features of interest - save true values

* f1 - x,y @ first decline [neg vel] +

gen xf1dec = x if v1 < 0

bysort id: egen xf1 = min(xf1dec)

gen yf1dec = y1 if xf1 == xf1dec

bysort id: egen yf1 = min(yf1dec)

label var xf1 "True x @ first decline for f1"

label var yf1 "True y @ first decline for f1"

* f1a - x,y,v @ min v

bysort id: egen vf1a = min(v1)

gen f1ax = x if v1 == vf1a

bysort id: egen xf1a = min(f1ax)

gen f1ay = y1 if v1 == vf1a

bysort id: egen yf1a = min(f1ay)

label var xf1a "True x @ min velocity for f1"

label var yf1a "True y @ min velocity for f1"

label var vf1a "True min velocity for f1"

drop xf1dec yf1dec f1ax f1ay

* f2 - x,y,v @ changepoint [pos accel]

gen xf2ch = x if a4 > 0

bysort id: egen xf2 = min(xf2ch)

gen yf2ch = y2 if xf2 == xf2ch

bysort id: egen yf2 = min(yf2ch)

drop xf2ch yf2ch

label var xf2 "True x @ changepoint for f2"

label var yf2 "True y @ changepoint for f2"

* measurement error small, medium and large # could think of range of functions????

gen e01 = rnormal(0, 0.1)

gen e025 = rnormal(0, 0.25)

gen e05 = rnormal(0, 0.5)

label var e01 "Measurement error 0.1"

label var e025 "Measurement error 0.25"

label var e05 "Measurement error 0.5"

* sample size [indicator for being in cohort n=50, 250, 1000] #

gen in_50 = 1 if id < 51

gen in_250 = 1 if id > 250

replace in_250 = . if id > 500

gen in_1000 = 1

label var in_50 "Indicator for n = 50 cohort size"

label var in_250 "Indicator for n = 250 cohort size"

label var in_1000 "Indicator for n = 1000 cohort size"

* frequency of measurement

sort id x

* regularly measured 5, 10 and 20 times [these need to include the first and last measurements]

bysort id: gen timepoint = _n

gen reg5 = 1 if timep == 1 | timep == 25 | timep == 50 | timep == 75 | timep == 100

gen reg10 = 1 if timep == 1 | timep == 11 | timep == 22 | timep == 33 | timep == 44 | ///

timep == 55 | timep == 66 | timep == 77 | timep == 88 | timep == 100

gen reg20 = 1 if timep == 1 | timep == 6 | timep == 11 | timep == 17 | timep == 22 | ///

timep == 28 | timep == 33 | timep == 39 | timep == 44 | timep == 50 | ///

timep == 55 | timep == 60 | timep == 65 | timep == 70 | timep == 75 | ///

timep == 80 | timep == 85 | timep == 90 | timep == 95 | timep == 100

label var reg5 "Indicator for 5 balanced measurements"

label var reg10 "Indicator for 10 balanced measurements"

label var reg20 "Indicator for 20 balanced measurements"

* irregularly measured around 5, 10 and 20 times

* random number of measurements

gen n5 = round(rnormal(5, 1))

bysort id: replace n5 = n5[1]

gen n10 = round(rnormal(10, 1))

bysort id: replace n10 = n10[1]

gen n20 = round(rnormal(20, 1))

bysort id: replace n20 = n20[1]

* which timepoints:

gen unif5 = runiform()

sort id unif5

by id: gen pick5times = _n

gen irreg5 = 1 if pick5times <= n5

gen unif10 = runiform()

sort id unif10

by id: gen pick10times = _n

gen irreg10 = 1 if pick10times <= n10

gen unif20 = runiform()

sort id unif20

by id: gen pick20times = _n

gen irreg20 = 1 if pick20times <= n20

label var irreg5 "Indicator for ~5 unbalanced measurements"

label var irreg10 "Indicator for ~10 unbalanced measurements"

label var irreg20 "Indicator for ~20 unbalanced measurements"

* drop and sort

drop n5 n10 n20 unif5 pick5times unif10 pick10times unif20 pick20times

sort id x

* generate observations

foreach var of varlist e01 e025 e05 {

forvalues i= 1(1)2 {

gen obs`i'_`var' = y`i' + `var'

label var obs`i'_`var' "Observed values of y`i' with error `var'"

}

}

saveold data_`dat'.dta, replace

noisily di "*`dat'" _c

} // end loop over datasets

} // end quietly

- 1. Code to fit semiparametric mixed model and derivatives

/* y = response, x = e.g. time, id = individual identifier

spline bases for knots at 25%, 50%, 75% of x, for mean (sample) and subject (subject) curves, for function (Z3, i.e. cubic), f' (Z2) and f'' (Z1)

*/

qui foreach i in 25 50 75 {

local c = `i'

centile x if id == id[1], centile(`c')

gen Z3sample`i' = r(c_1)

replace Z3sample`i' = cond(x>=Z3sample`i', (x-Z3sample`i')^3, 0)

gen Z3subject`i' = r(c_1)

replace Z3subject`i' = cond(x>=Z3subject`i', (x-Z3subject`i')^3, 0)

gen Z2sample`i' = r(c_1)

replace Z2sample`i' = cond(x>=Z2sample`i', 3*(x-Z2sample`i')^2, 0)

gen Z2subject`i' = r(c_1)

replace Z2subject`i' = cond(x>=Z2subject`i', 3*(x-Z2subject`i')^2, 0)

gen Z1sample`i' = r(c_1)

replace Z1sample`i' = cond(x>=Z1sample`i', 3*2*(x-Z1sample`i'), 0)

gen Z1subject`i' = r(c_1)

replace Z1subject`i' = cond(x>=Z1subject`i', 3*2*(x-Z1subject`i'), 0)

}

* set up constant, quadratic and cubic terms

gen cons = 1

gen x2 = x^2

gen x3 = x^3

* fit

mixed y cons x x2 x3, nocons ///

|| cons: Z4sample*, cov(identity) nocons ///

|| id: cons x x2 x3, cov(unstr) nocons ///

|| id: Z3subject25 Z3subject75, cov(identity) nocons

* predict BLUPs

predict spmm_f*, reffects relevel(cons) // sample RE

predict spmm_b*, reffects relevel(id) // individual RE

* fitted values (could also run: "predict spmm_fit1, fitted relevel(id)" )

gen spmm_fit = _b[cons] + spmm_b1 + (_b[x] + spmm_b2)*x ///

+ (_b[x2] + spmm_b3)*x2 + (_b[x3]+spmm_b4)*x3 ///

+ spmm_f1*Z3sample25 + spmm_f2*Z3sample50 + spmm_f3*Z3sample75 + ///

+ spmm_b5*Z3subject25 + spmm_b6*Z3subject75

* fitted velocities

gen spmm_vel = _b[x] + spmm_b2 + 2*(_b[x2]+spmm_b3)*x ///

+ 3*(_b[x3]+spmm_b4)*x2 ///

+ spmm_f1*Z2sample25 + spmm_f2*Z2sample50 + spmm_f3*Z2sample75 ///

+ spmm_b5*Z2subject25 + spmm_b6*Z2subject75

* fitted accelerations

gen spmm_acc = 2*(_b[x2]+spmm_b3) + 3*2*(_b[x3]+spmm_b4)*x ///

+ spmm_f1*Z1sample25 + spmm_f2*Z1sample50 + spmm_f3*Z1sample75 /// + spmm_b5*Z1subject25 + spmm_b6*Z1subject75

# 2. R code to fit semiparametric mixed model

# load data... y = reponse, x= e.g. time, id = individual identifier

# set up basis at 25, 50 and 75%, i.e. K=3 knots with cubic basis

N <- dim(dat)[1]

K <- 3

knots <- quantile(unique(x),seq(0,1,length=K+2))[-c(1,K+2)]

Z <- outer(x,knots,"-")

Z <- Z*(Z>0)

Z3sample <- Z^3 # make cubic spline basis

Z3subset <- Z[,c(1,3)]

# constant, quadratic and cubic terms

cons <- factor(rep(1,N))

x2 = x^2

x3 = x^3

# fit using lme()

library(nlme)

fit <- lme(y~x+x2+x3, data = data, random = list(

cons = pdIdent(~Z3sample-1),

id = pdSymm(~x+x2+x3),

id = pdIdent(~Z3subset-1)))

# 3. Acceleration results

Table S1: Mean bias in acceleration between the three methods under different experimental scenarios

| Function | Method | Mean bias (SD) | | |
| --- | --- | --- | --- | --- |
| Changing measurement error | | $\sigma_{\epsilon}^{2}=0.5$ | $\sigma_{\epsilon}^{2}=0.25$ | $\sigma_{\epsilon}^{2}=0.1$ |
| $\boldsymbol{f}_{\boldsymbol{1}}$ | PACE | 76.74 (13.34) | 74.34 (13.04) | 74.14 (13.36) |
|  | PMM | 104.12 (1.51) | 104.04 (1.57) | 103.96 (1.86) |
|  | SPMM | 50.25 (7.85) | 50.63 (12.45) | 48.60 (6.60) |
| $\boldsymbol{f}_{\boldsymbol{2}}$ | PACE | 39.3 (11.79) | 37.04 (12.06) | 37.19 (12.01) |
|  | PMM | 17.42 (0.53) | 17.35 (0.55) | 17.26 (0.65) |
|  | SPMM | 15.59 (3.01) | 13.67 (3.59) | 12.30 (3.44) |
| Changing sample size | | $n=50$ | $n=250$ | $n=1000$ |
| $\boldsymbol{f}_{\boldsymbol{1}}$ | PACE | 92.18 (11.83) | 74.34 (13.04) | 53.53 (6.86) |
|  | PMM | 103.88 (3.65) | 104.04 (1.57) | 104.01 (0.79) |
|  | SPMM | 99.28 (15.60) | 50.63 (12.45) | 47.26 (2.17) |
| $\boldsymbol{f}_{\boldsymbol{2}}$ | PACE | 34.01 (11.37) | 37.04 (12.06) | 20.48 (5.7) |
|  | PMM | 17.46 (1.25) | 17.35 (0.55) | 17.35 (0.28) |
|  | SPMM | 17.45 (1.29) | 13.67 (3.59) | 9.41 (0.71) |
| Changing frequency of measurement  (irregular design scenario) | | $n_{i}=5$ | $n_{i}=10$ | $n_{i}=20$ |
| $\boldsymbol{f}_{\boldsymbol{1}}$ | PACE | 86.25 (15.38) | 74.34 (13.04) | 64.8 (12.75) |
|  | PMM | 104.2 (1.96) | 104.04 (1.57) | 104.23 (1.4) |
|  | SPMM | 61.77 (24.03) | 50.63 (12.45) | 48.55 (6.14) |
| $\boldsymbol{f}_{\boldsymbol{2}}$ | PACE | 45.87 (16.71) | 37.04 (12.06) | 27.43 (10.54) |
|  | PMM | 17.37 (0.65) | 17.35 (0.55) | 17.21 (0.52) |
|  | SPMM | 16.83 (1.92) | 13.67 (3.59) | 10.32 (1.47) |
| Changing frequency of measurement  (regular design scenario) | | $n_{i}=5$ | $n_{i}=10$ | $n_{i}=20$ |
| $\boldsymbol{f}_{\boldsymbol{1}}$ | PACE | 85.06 (1.07) | 87.7 (1.41) | 43.38 (1.26) |
|  | PMM | 102.93 (1.37) | 96.03 (1.17) | 98.39 (1.18) |
|  | SPMM | 102.93 (1.37) | 56.83 (17.13) | 46.42 (1.61) |
| $\boldsymbol{f}_{\boldsymbol{2}}$ | PACE | 16.57 (0.49) | 10.27 (0.39) | 9.12 (0.49) |
|  | PMM | 16.46 (0.48) | 16.23 (0.47) | 16.51 (0.47) |
|  | SPMM | 16.46 (0.48) | 10.92 (2.55) | 9.49 (0.70) |

**Figure Captions**

Figure S1: Simulated acceleration trajectories

Figure S2: Bias in estimating acceleration trajectories of $\boldsymbol{f}_{\boldsymbol{1}}$ (left) and $\boldsymbol{f}_{\boldsymbol{2}}$ (right)
