# Supplementary figures and images for: Derivative estimation for longitudinal data analysis: Examining features of blood pressure measured repeatedly during pregnancy
Source: Stat Med. 2018 May 20;37(19):2836–54. doi: 10.1002/sim.7694 (PMC6099422; doi:10.1002/sim.7694)

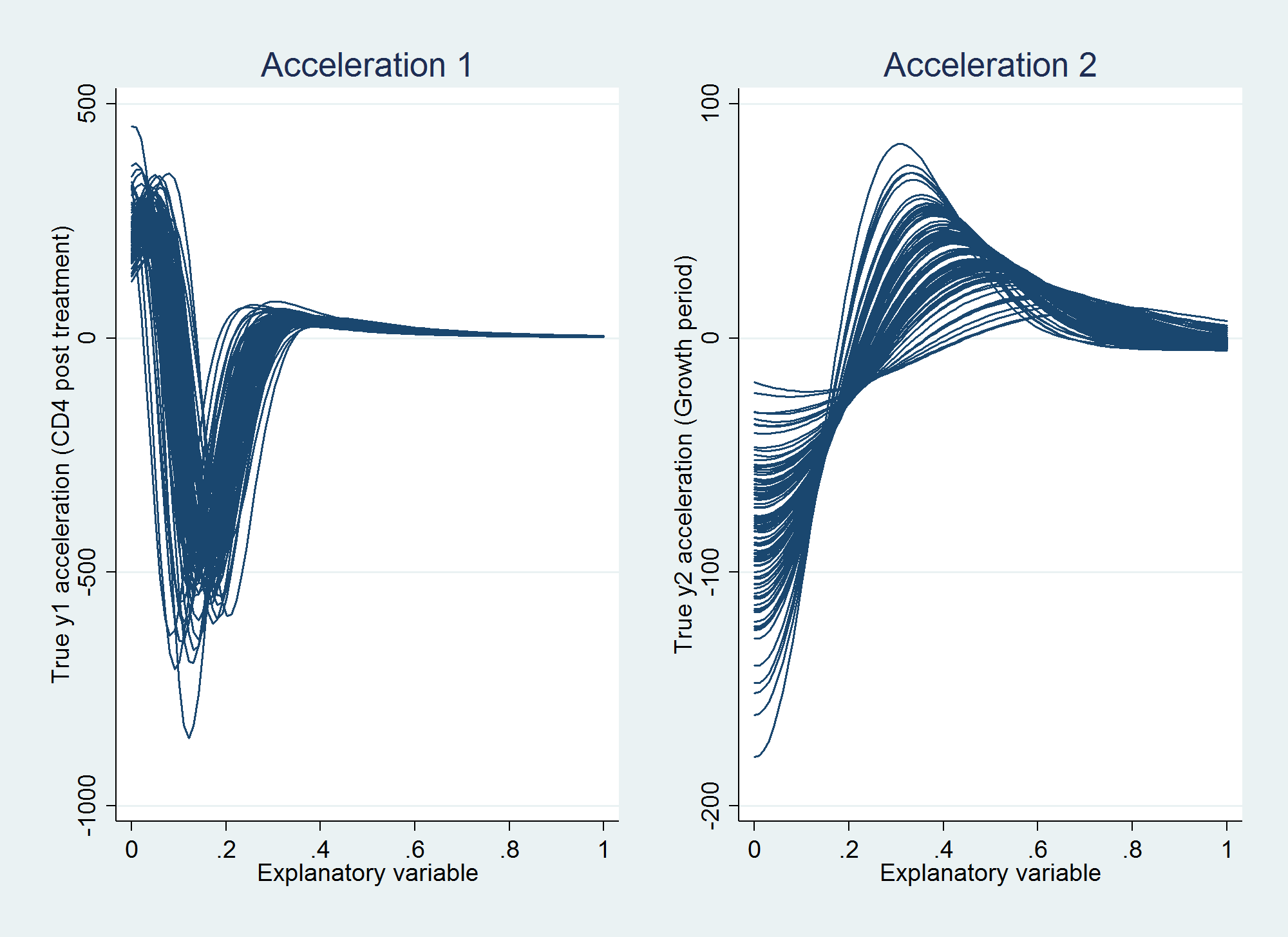

Supplement: Supplementary file 2 — Supporting info item [file SIM-37-2836-s002.tif]

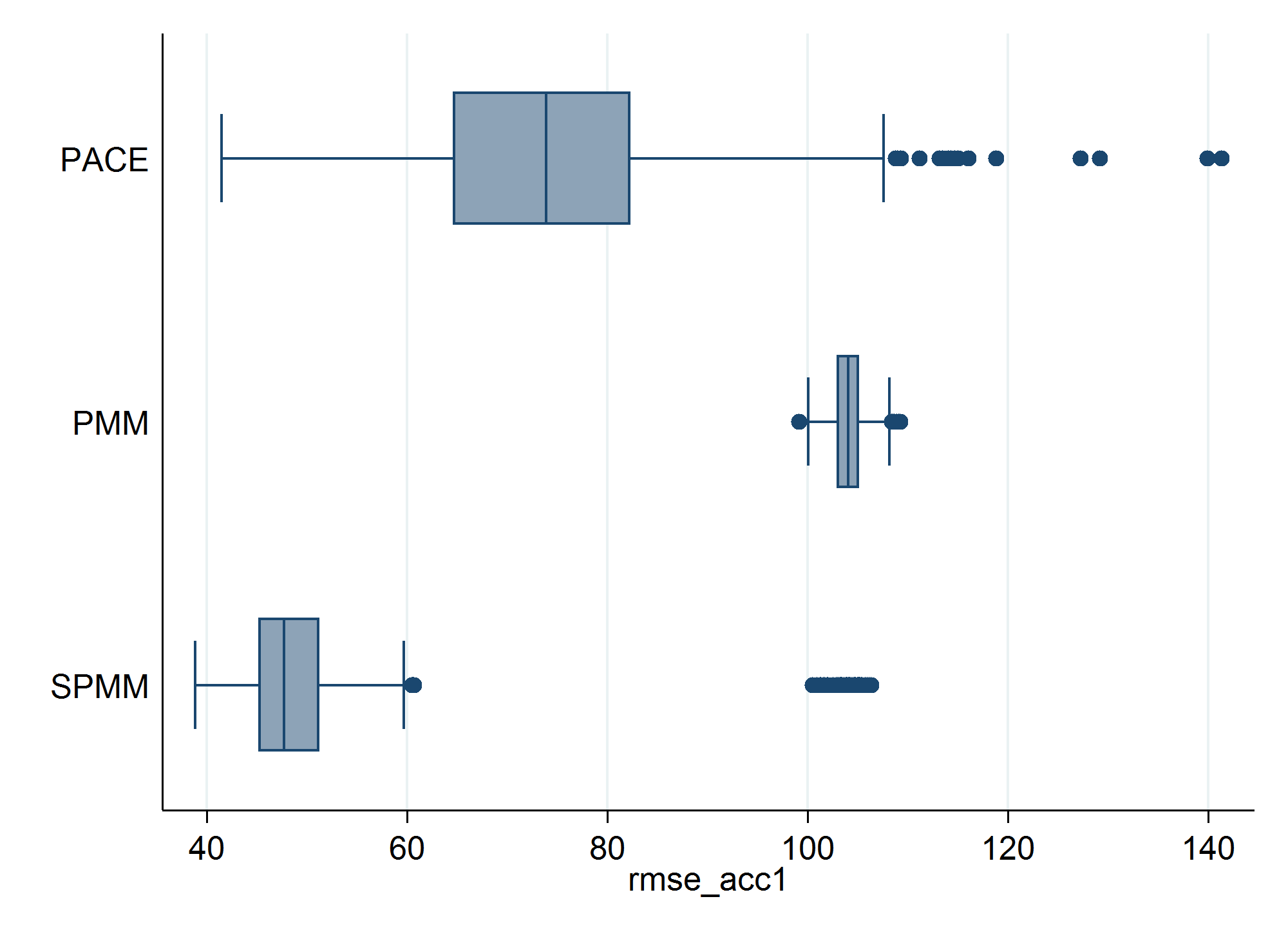

Supplement: Supplementary file 3 — Supporting info item [file SIM-37-2836-s003.tif]

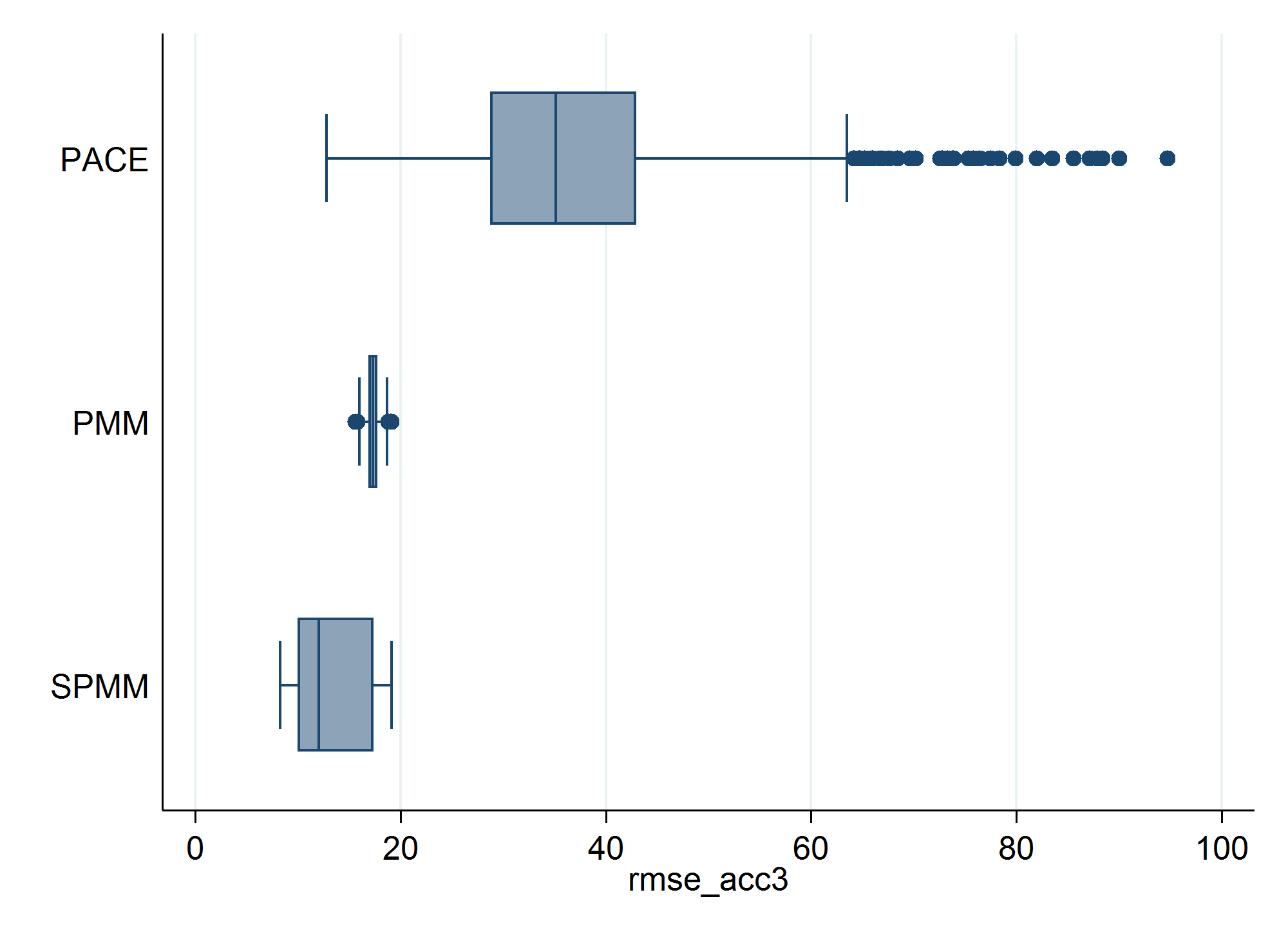

Supplement: Supplementary file 4 — Supporting info item [file SIM-37-2836-s004.tif]
